# ﻿Assembly and comparative analysis of the complete mitochondrial genome of *Daedaleopsissinensis* (Polyporaceae, Basidiomycota), contributing to understanding fungal evolution and ecological functions

**DOI:** 10.3897/imafungus.16.141288

**Published:** 2025-02-17

**Authors:** Jin-Xin Ma, Hai-Jiao Li, Can Jin, Hao Wang, Lu-Xin Tang, Jing Si, Bao-Kai Cui

**Affiliations:** 1 State Key Laboratory of Efficient Production of Forest Resources, School of Ecology and Nature Conservation, Beijing Forestry University, Beijing 100083, China Beijing Forestry University Beijing China; 2 National Institute of Occupational Health and Poison Control, Chinese Center for Disease Control and Prevention, Beijing 100050, China National Institute of Occupational Health and Poison Control, Chinese Center for Disease Control and Prevention Beijing China

**Keywords:** Comparative genomics, mitochondrial genetics, phylogenetics, species evolution, wood-decaying fungi

## Abstract

*Daedaleopsissinensis* is a crucial wood-decaying fungus with significant lignocellulose-degrading ability, which plays a vital role in the material cycle and energy flow of forest ecosystems. However, the mitochondrial genome of *D.sinensis* has not yet been revealed. In the present study, the complete mitochondrial genome of *D.sinensis* was assembled and compared with related species. The mitochondrial genome spans 69,155 bp and has a GC content of 25.0%. It comprises 15 protein-coding genes (PCGs), 26 transfer RNA genes, two ribosomal RNA genes and one DNA polymerase gene (*dpo*). Herein, we characterised and analysed the codon preferences, variation and evolution of PCGs, repeats, intron dynamics, as well as RNA editing events in the *D.sinensis* mitochondrial genome. Further, a phylogenetic analysis of *D.sinensis* and the other 86 Basidiomycota species was performed using mitochondrial genome data. The results revealed that four species, *D.confragosa*, *D.sinensis*, *D.nitida* and *Fomesfomentarius*, were grouped in a closely-related cluster with high support values, indicating that a close phylogenetic relationship existed between *Daedaleopsis* and *Fomes*. This study reported on the initial assembly and annotation of the mitochondrial genome of *D.sinensis*, which greatly improved the knowledge of the fungus. These results contribute to the limited understanding of the mitochondrial repository of wood-decaying fungi, thereby laying the foundation for subsequent research on fungal evolution and ecological functions.

## ﻿Introduction

**Figure 1.**
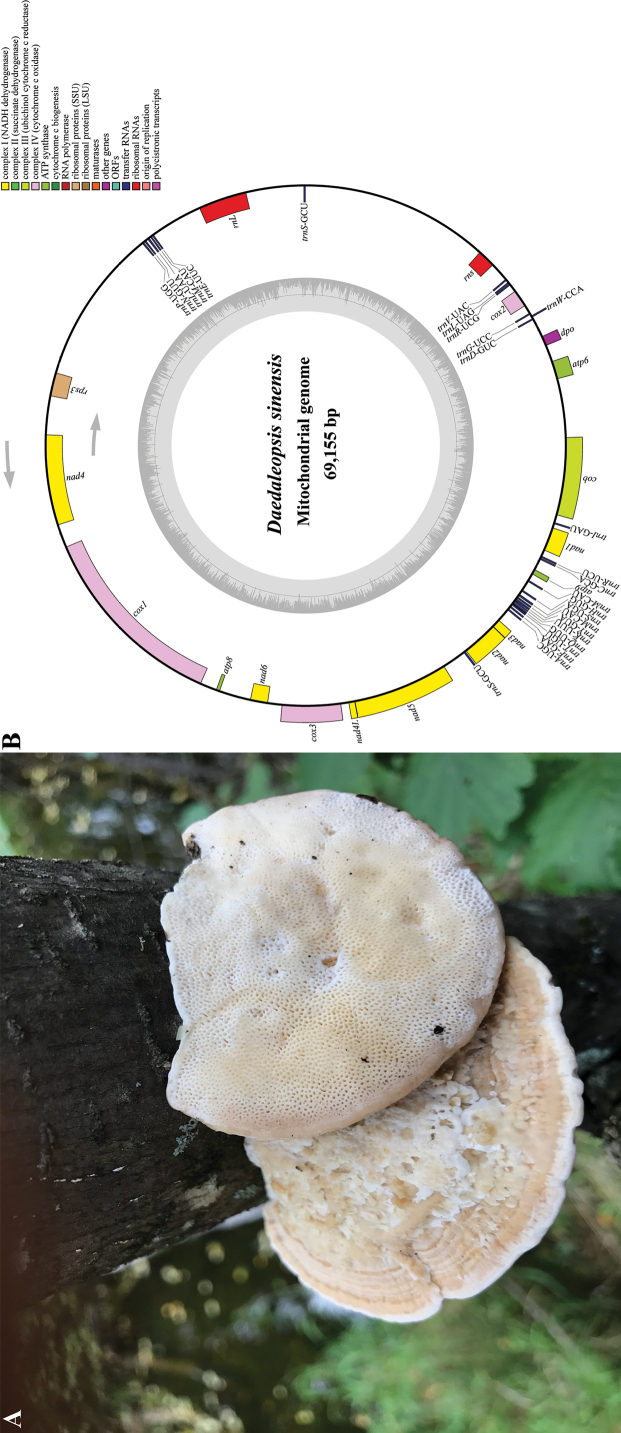
**A** Reference image of *Daedaleopsissinensis*. The sample was collected from Changbai Mountain, Antu County, Yanbian Prefecture, Jilin Province, China on 27 Sep 2019 and photographed by Hai-Jiao Li **B** a circular map of the assembled mitochondrial genome of *Daedaleopsissinensis*, which comprises 15 PCGs, 26 tRNAs, two rRNA genes and one *dpo* gene. The inner ring indicates the GC content. The genes encoded on the reverse and forward strands are shown inside and outside the circles, respectively.

Wood-decaying fungi are essential components of forest ecosystems. It is possible to re-introduce dead branches and decayed wood degraded by wood-decaying fungi into nature. Retaining these degraded wood residues in the soil can increase its aeration and water-holding capacity, promote the formation of ectomycorrhizal roots and increase the nitrogen-fixing capacity of certain microorganisms. Wood-decaying fungi thus participate in the material cycle and energy flow of ecosystems, promote metabolism and maintain a dynamic equilibrium (Larsen et al. 1979, 1982). In addition, several lignocellulose-degrading enzymes secreted by wood-decaying fungi have been shown to have significant ecological and economic value. *Daedaleopsissinensis* is a wood-decaying fungus that causes white rot in wood. *D.sinensis* (Lloyd) Y.C. Dai (1996: 90) is a member of the Polyporaceae family in the Basidiomycota division, found in northern China and far eastern Russia (Amur, Khabarovsk) (Lloyd 1922; Dai 1996; Núñez and Ryvarden 2001). Fig. 1A presents a reference image of the species. *D.sinensis* attacks the heartwood of living hardwood trees, primarily *Betula* and *Alnus*, causing white rot and exerting a significant lignin-degrading capacity (Ma et al. 2024). The morphological characteristics of *D.sinensis* include triquetrous basidiocarps, sinuous pores and a cream to pale ochraceous, glabrous pileus featuring prominent warts and scrupose protuberances. When mature, it has cream to pale buff context and tubes and highly lacerated pores (Núñez and Ryvarden 2001; Li et al. 2016).

Mitochondria play vital roles in eukaryotic cells, particularly in respiratory metabolism and energy production. The in-depth study of the role of these organelles is required to provide a more comprehensive and thorough understanding of fungal evolution, secondary metabolism, biodegradation, artificial domestication and other related topics, due to their small size, rapid evolution and significant involvement in the growth and development of mitochondrial genomes (Basse 2010; Miao et al. 2022). The mitochondrial genome is used to analyse the genetic and evolutionary relationships of organisms. It has a higher mutation rate than nuclear genes, facilitating the discovery of subtle gene changes and making it easier to analyse the differences in the same genes in different species, thus determining their affinities (Han et al. 2020). The size and evolution rate of the fungal mitochondrial genome are comparable to those of the mitochondrial genomes of animals and plants. Most fungal mitochondrial genes are conserved, although the number and distribution of gene introns are highly varied, while the order of genes can also differ (Paquin et al. 1997; Aguileta et al. 2014). Despite fungi belonging to a lineage closer to that of animals, the recombination signals in fungal mitochondria are more similar to those identified in plant mitochondria (Lang et al. 2007). Although there has been a rapid development of high-throughput technologies in recent years, the study of mitochondrial genomes in fungi is not yet sufficiently extensive (Mardanov et al. 2014; Song et al. 2020). The number of fungal mitochondrial genomes available in the National Center of Biotechnology Information (NCBI) database is far smaller than that of animals (https://www.ncbi.nlm.nih.gov/genome/browse#!/organelles/). Further, fungal mitochondrial genomes are significantly less studied than those of plants and animals, despite presenting a significant resource to understand and elucidate the evolution of organelle genomes (Aguileta et al. 2014). According to the mitochondrial data available in the database, the mitochondrial genomes of different fungal species differ in size, composition, gene order, repeat composition and content and intron type and number, even amongst closely-related species. The mitochondrial genomes of most fungi include a conserved set of protein-coding genes (PCGs), including three ATP synthase subunit genes (*atp6*, *atp8* and *atp9*), one cytochrome b gene (*cob*), three cytochrome C subunit genes (*cox1*, *cox2* and *cox3*), seven NADH dehydrogenase subunit genes (*nad1*, *nad2*, *nad3*, *nad4*, *nad4L*, *nad5* and *nad6*) and a non-core gene, the ribosomal protein S3 gene (*rps3*), which plays an important role in maintaining cellular energy supply and homeostasis (Osiewacz 2002; Chatre and Ricchetti 2014; Ma et al. 2022).

Within *Daedaleopsis*, the mitochondrial genomes of only two species, *D.confragosa* and *D.nitida*, have been reported. This study sequenced and analysed the mitochondrial genome of *D.sinensis* and investigated its phylogenetic relationship with related species. These results can enrich the fungal mitochondrial database and provide crucial reference data and important clues for further research on fungal phylogenetics and evolutionary relationships and the subsequent utilisation of wood-decaying fungi.

## ﻿Methods

### ﻿Fungal material and sequencing

The strain Si 85 used in this study was isolated from the fruiting bodies of *D.sinensis* collected from a fallen angiosperm branch in Changbai Mountain, Antu County, Yanbian Prefecture, Jilin Province, China (altitude: 574 m; 42°31'31"N, 128°3'15"E). The specimen is currently deposited at the Herbarium of School of Ecology and Nature Conservation of Beijing Forestry University in China. Mycelia were harvested following incubation at 28 °C for 7–14 days on potato dextrose agar media (20.0 g/l agar, 20.0 g/l glucose, 200.0 g/l potato and 1.0 l distilled water). Genomic DNA was extracted from the mycelia using the cetyltrimethyl ammonium bromide method (Watanabe et al. 2010). Whole-genome sequencing was performed using the PacBio Sequel and MGISEQ2000 platforms (Nextomics Biosciences Co., Ltd.), according to the manufacturer’s instructions, using GrandOmics (https://www.grandomics.com/, Wuhan, China). The raw reads were subjected to quality control to acquire clean data and used to assemble the mitochondrial genome with GetOrganelle v.1.7.7.0 (Jin et al. 2020). The genome sequence was assembled on Bandage (Wick et al. 2015) to verify its ring structure. Subsequently, the sequence was automatically annotated with MITOS (Donath et al. 2019), after which it was manually corrected. The assembled and complete mitochondrial genome was deposited in GenBank under the accession number NC_085747.

### ﻿Structural annotation of the mitochondrial genome

Using published fungal mitochondrial sequences, the PCGs and ribosomal RNA (rRNA) genes of *D.sinensis* were annotated and the sequences of *D.sinensis* matched those of closely-related species. The transfer RNA (tRNA) genes were annotated using tRNAscan-SE (http://lowelab.ucsc.edu/tRNAscan-SE/) (Chan and Lowe 2019). A mitochondrial genome map of *D.sinensis* was drawn using the OGDRAW web server (https://chlorobox.mpimp-golm.mpg.de/OGDraw.html) (Greiner et al. 2019).

### ﻿Elucidation of sequencing characteristics and intron analysis

CodonW v.1.4.4 (Sharp and Li 1986) was applied to calculate the relative synonymous codon usage (RSCU) values and the data generated above were collated and visualised in R v.4.3.1. The mitochondrial repeats were divided into three main parts for analysis, from which MISA v.2.1 (Thiel et al. 2003; Beier et al. 2017), TRF (Benson 1999) and REPuter (Kurtz et al. 2001) were used to detect simple sequence repeats (SSRs), tandem repeats and interspersed repeats, respectively. The identified repeats were visualised using Circos v.0.69.9 (Krzywinski et al. 2009). The AT and GC skews of PCGs in *Daedaleopsis* were calculated using the following formulas: AT-skew = (A–T)/(A+T) and GC-skew = (G–C)/(G+C). R was used to visualise PCG length variation, GC content, AT skew and GC skew. The three mitochondrial genomes of *Daedaleopsis* species were aligned using MAFFT v.7.471 (Katoh and Standley 2013; Rozewicki et al. 2019) and the *Ka* and *Ks* values of the genes, representative of the mean numbers of non-synonymous and synonymous substitutions in each non-synonymous and synonymous sites, were determined using KaKs_Calculator v.2.0 (https://sourceforge.net/projects/kakscalculator2/) via the MLWL calculation method (Wang et al. 2010). The Kimura-2-parameter (K2P) genetic distances were calculated by MEGA v.11 (Tamura et al. 2021). The GenBank IDs for the mitochondrial genomes of the other two *Daedaleopsis* species were as follows: *D.confragosa*: NC_084114; *D.nitida*: NC_087776.

Subsequently, the correlation between intron number and mitochondrial genome size was analysed using the Pearson correlation coefficient. Further, the introns of *cox1* genes in the mitochondrial genomes of 23 species in Polyporaceae were classified into different position classes (Pcls), according to the method described by Férandon et al. (2010). The *cox1* genes in the mitochondrial genomes of 23 Polyporaceae fungi were aligned with those of *Ganodermacalidophilum* using Clustal W (Thompson et al. 1994; Li et al. 2019b). Each Pcl comprises introns inserted at the same location in the *cox1* gene. The same Pcls usually have high sequence similarity.

### ﻿Phylogenetics of mitochondrial genomes

The NCBI website was used to search for publicly available mitochondrial genome data for fungi, selecting suitable species according to fungal taxonomy categories. We selected mitochondrial data of fungi belonging to Basidiomycota for analysis; information on the 87 varieties is included in Suppl. material 1: table S1. The gene sequences of the PCGs were extracted and imported into MAFFT v.7.471 for multiple sequence alignment (Katoh and Standley 2013). MACSE v.2 (Ranwez et al. 2018) and Gblock (Talavera and Castresana 2007) were used to optimise and trim the MAFFT alignment results, respectively. Multiple genes were concatenated and PartitionFinder v.2.1.1 (Lanfear et al. 2017) was applied to identify optimal partitioning strategies and evolutionary models. The phylogenetic relationships of the mitochondrial genomes of 87 Basidiomycota species were determined using IQ-tree and MrBayes v.3.2, following the Bayesian Inference (BI) and Maximum Likelihood (ML) methods (Ronquist et al. 2012; Nguyen et al. 2015). All analyses were conducted within PhyloSuite (Zhang et al. 2020). The results were visualised and embellished on the iTOL website (https://itol.embl.de/) (Letunic and Bork 2007).

### ﻿Prediction of RNA editing sites

The Deepred-mt tool, which is based on a convolutional neural network model, was applied to predict cytidine-to-uridine RNA editing events in the mitochondrial genome of *D.sinensis* (Edera et al. 2021). Deepred-mt’s predictions were generally regarded as reliable and data with a probability value higher than 0.9 for further analysis were selected. The high threshold of 0.9 ensures confidence, thereby improving the accuracy and reliability of RNA editing analyses in mitochondrial genomes.

### ﻿Collinearity analysis

The mitochondrial genomes of nine closely-related species were analysed using BLAST (Chen et al. 2015) and a multiple synteny plot was generated and visualised using MCScanX software (Wang et al. 2012). Mauve v.2.4.0 was applied to analyse and visualise the collinearity and rearrangement between homologous regions of the three *Daedaleopsis* species (Darling et al. 2004).

### ﻿Abbreviations

**BI** Bayesian Inference

**BPP** Bayesian posterior probability

**K2P** Kimura-2-parameter

**ML** Maximum Likelihood

**PCG** Protein-coding gene

**Pcl** Position class

**rRNA** Ribosomal RNA

**RSCU** Relative synonymous codon usage

**SSR** Simple sequence repeat

**tRNA** Transfer RNA

## ﻿Results

### ﻿Features of the mitochondrial genome of *Daedaleopsissinensis*

Fig. 1A presents a representative image of the fruiting body of *D.sinensis* in the wild. We newly assembled the complete mitochondrial genome of *D.sinensis* and compared and analysed it with the publicly-available mitochondrial genomes of *D.confragosa* and *D.nitida*. The mitochondrial genome of *D.sinensis* is a closed circular DNA molecule 69,155 bp in length, with a GC content of 25.0%. The GC content of *D.confragosa* and *D.nitida* were similar to *D.sinensis*, at 25.2% and 25.3%, respectively. The mitochondrial genome of *D.sinensis* comprises 15 PCGs, two rRNA genes, 26 tRNA genes and one DNA polymerase gene (*dpo*) (Fig. 1B; Suppl. material 1: table S2). An entire set of PCGs in *D.sinensis* was detected, including *atp6*, *atp8*, *atp9*, *cob*, *cox1*, *cox2*, *cox3*, *nad1*, *nad2*, *nad3*, *nad4*, *nad4L*, *nad5*, *nad6* and *rps3* genes. The two rRNA genes identified were the small (*rns*) and large subunit ribosomal RNA (*rnl*), which encode the small and large RNA molecules of the mitochondrial ribosome, respectively. D.sinensisalso containsadpo gene, which is known to have a plasmid origin, encodes a DNA-directed DNA polymerase and comprises mitochondrial plasmid-related genes. The proportion of AT nucleotides in the mitochondrial genome of *D.sinensis* was greater than that of GC nucleotides.

### ﻿RNA genes

Each mitochondrial genome of the three *Daedaleopsis* species contained two rRNA genes: *rns* and *rnl* (Suppl. material 1: table S1). The average lengths of *rnl* and *rns* in the three species were 2,293 and 1,265 bp, respectively. The 26 tRNAs contained in the *D.sinensis* were folded into classic clover structures (Suppl. material 2: fig. S1). The 26 tRNA genes identified in *D.sinensis* encode 20 standard amino acids and range in length from 71 to 88 bp. Owing to its sizeable extra arm, the *trnS* gene had the largest volume of all the detected tRNA genes. Due to the secondary structure folding process of tRNA, base mismatches occur. The tRNA genes shared by the three *Daedaleopsis* species varied in location between the three mitochondrial genomes. A total of 39 base mismatches were identified in tRNA genes in the mitochondrial genome of *D.sinensis*, amongst which 38 were G-U mismatches and one was an A-C mismatch. The mismatched base pairs were distributed in different parts of the tRNAs. Of the tRNA genes shared by the three *Daedaleopsis* species, there are five variable sites, shown in red in Suppl. material 2: fig. S1. The structure in blue depicts one tRNA in the *D.nitida* that is completely distinct from the other *Daedaleopsis* species.

### ﻿Codon usage of PCGs

**Figure 2.**
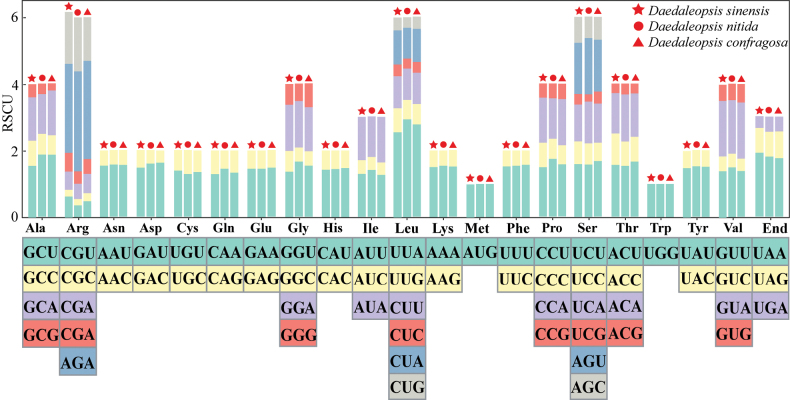
Bar chart of RSCU values, which represent the specific codon frequency compared with the expected frequency of uniform synonymous codon usage, with the x-axis representing the codon families. The box on the bottom represents all the codons encoding each amino acid.

The RSCU of the three *Daedaleopsis* species were nearly identical, with only minimal differences. The most used codons for each amino acid are depicted in Fig. 2. Arginine (Arg), leucine (Leu) and serine (Ser) were the most frequent amino acids, whereas methionine (Met) and tryptophan (Trp) were less common. Amongst the 64 codons in PCGs, an RSCU value equal to 1 indicates no preference for the amino acid, while an RSCU value greater than 1 indicates a preference for the amino acid. The RSCU values for Met (AUG) and Trp (UGG) were 1. Moreover, 29 codons had RSCU values greater than 1 and were classified as the high-frequency codons. The highest RSCU value was 2.67 for Arg (AGA), indicating a clear preference for AGA, followed by Leu (UUA) and the end codon (UAA), with RSCU values of 2.56 and 1.92, respectively (Suppl. material 1: table S3).

### ﻿Variations, genetic distances and evolutionary rates of PCGs

**Figure 3.**
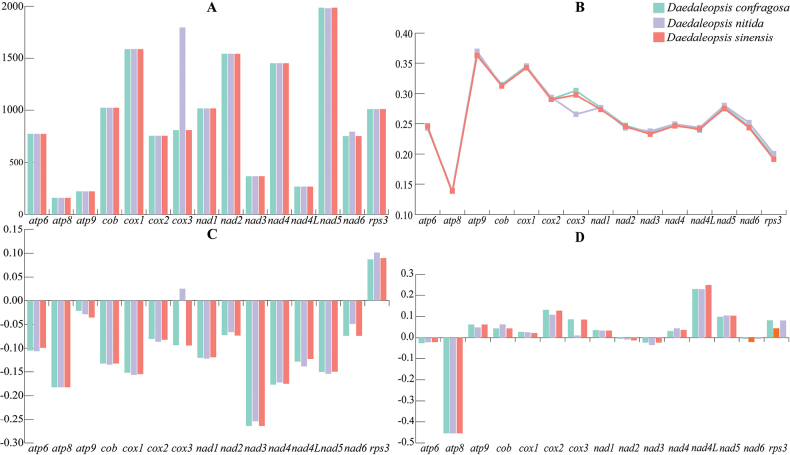
Variations in the length and base composition of individual PCGs in the mitochondrial genomes of the three *Daedaleopsis* species **A**PCG length variation **B** GC content of the different PCGs **C** AT skew **D** GC skew.

The mitochondrial genome of *D.sinensis* contains 15 PCGs totalling 13,725 bp in length or 19.85% of the total length of the mitochondrial genome. The shortest (*atp8*) was 159 bp, while the longest (*nad5*) was 1,986 bp, indicating significant length disparities. Two of the 15 PCGs detected (*cox3* and *nad6*) showed obvious variations in length amongst the three *Daedaleopsis* species analysed (Fig. 3A). The initiation and termination codons for the 15 PCGs were ATG and TAA, respectively. PCGs were classified into four categories according to their various functions. These genes included three ATP synthase-related genes (*atp6*, *atp8* and *atp9*), four cytochrome-related genes (*cox1*, *cox2*, *cox3* and *cob*), seven NADH dehydrogenase-related genes (*nad1*, *nad2*, *nad3*, *nad4*, *nad4L*, *nad5* and *nad6*) and one ribosomal protein-encoding gene (*rps3*) (Suppl. material 1: table S2). The length of the *cox3* gene varied the most amongst *Daedaleopsis* species, with *D.nitida* having the longest *cox3* gene. With the exception of *atp8*, which was completely unchanged in all three *Daedaleopsis* species, and *cox3*, which was significantly different, the GC contents of the other PCGs did not differ remarkably (Fig. 3B). There are fewer noticeable differences in the AT and GC skews of 15 PCGs in the three *Daedaleopsis* species, with the majority showing negative AT skews and positive GC skews (Fig. 3C, D). The AT skew in 15 PCGs varied amongst the three *Daedaleopsis* species, indicating that A/T mutations occurred more frequently in PCGs. Most PCGs were positive for GC skew, suggesting that most PCGs tend to evolve towards being G-rich rather than C-rich in the leading strand of PCGs.

**Figure 4.**
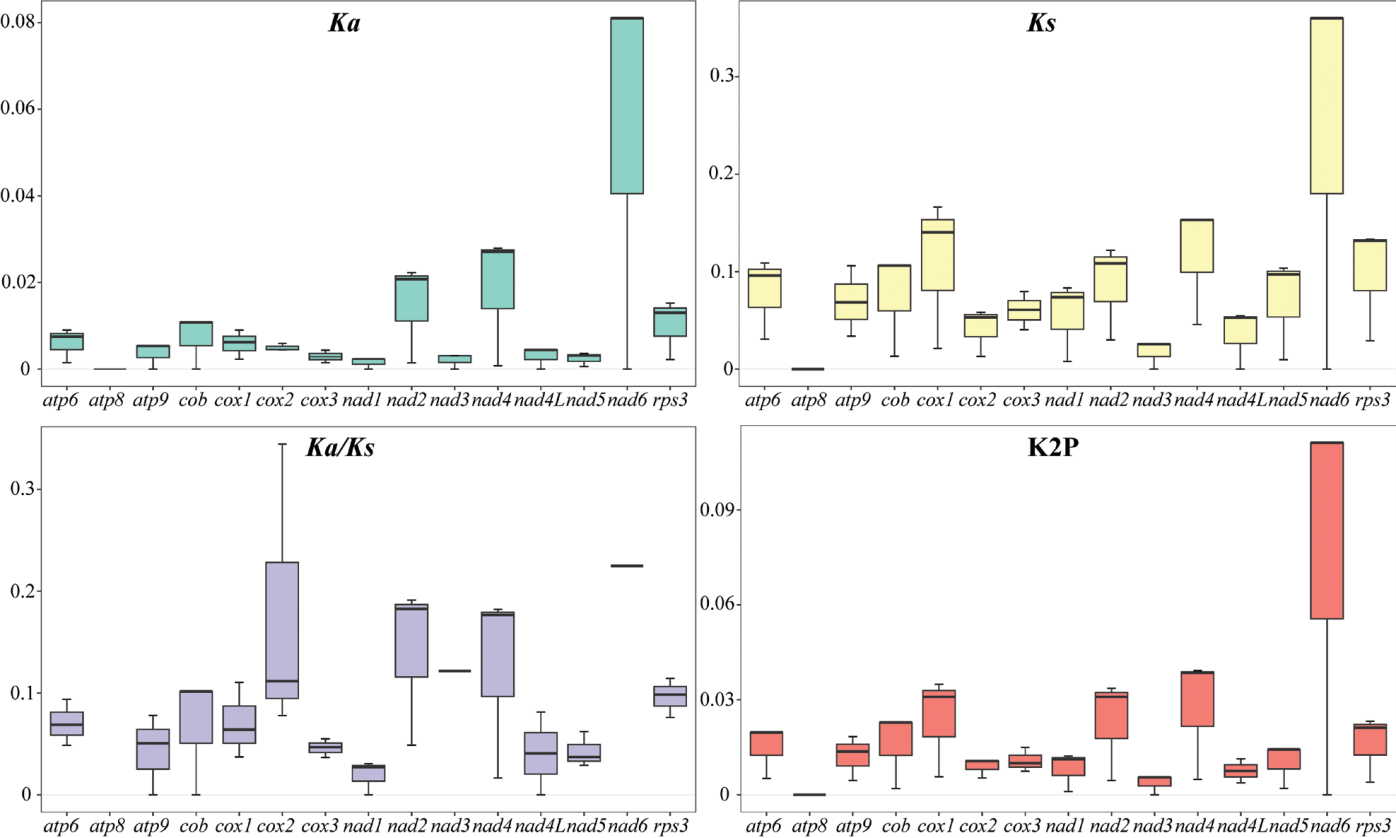
Genetic analysis of the 15 PCGs conserved in the mitochondrial genomes of the three *Daedaleopsis* species. *Ka*, the mean number of non-synonymous substitutions per non-synonymous site; *Ks*, the mean number of synonymous substitutions per synonymous site; K2P, genetic distance according to the Kimura-2-parameter.

Since the mitochondrial genomes of the three *Daedaleopsis* species contain 14 core PCGs and one *rps3* gene, here 15 PCGs were used to calculate the K2P genetic distances and substitution rates (Fig. 4). While *nad6* presented the highest average non-synonymous and synonymous replacement rates, *atp8* presented the lowest average *Ka* and *Ks* rates, both equal to zero. In addition, *atp8* in the three species were identical in size and arrangement. Therefore, a comparison of the *atp8* gene sequences revealed that *atp8* was highly conserved across the three *Daedaleopsis* species. All the PCGs had *Ka*/*Ks* values of less than 1.0, implying that the genes of *Daedaleopsis* were under pressure from purifying selection. The K2P genetic distance amongst the *nad6* genes was the greatest, followed by that amongst the *nad4* genes, indicating significant divergence during evolution. Amongst the mitochondrial genomes of the three *Daedaleopsis* species, the *atp8* and *nad3* genes presented the lowest average K2P genetic distances, suggesting high levels of conservation.

### ﻿Intron dynamics of *cox1* genes

**Figure 5.**
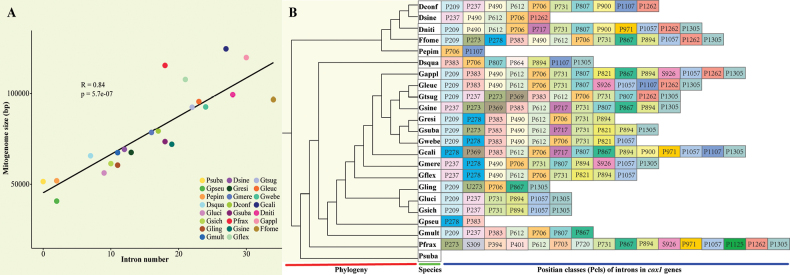
**A** Pearson correlation analyses between the number of introns and sizes of mitochondrial genomes of 23 *Polyporaceae* species **B**Pcl information of *cox1* genes. Introns in *cox1* genes of 23 mitochondrial genomes were classified into different Pcls using the *cox1* gene of *Ganodermacalidophilum* as the reference. The phylogenetic positions of the 23 species were established using the BI and ML methods, based on concatenated mitochondrial genes. The species indicated by the abbreviations are as follows: Dconf, *Daedaleopsisconfragosa*; Dsine, *D.sinensis*; Dniti, *D.nitida*; Ffome, *Fomesfomentarius*; Pepim, *Porogrammeepimiltina*; Dsqua, *Dichomitussqualens*; Gappl, *G.applanatum*; Gleuc, *G.leucocontextum*; Gtsug, *G.tsugae*; Gsine, *G.sinensis*; Gresi, *G.resinaceum*; Gsuba, *G.subamboinense*; Gwebe, *G.weberianum*; Gcali, *G.calidophilum*; Gmere, *G.meredithae*; Gflex, *G.flexipes*; Gling, *G.lingzhi*; Gluci, *G.lucidum*; Gsich, *G.sichuanense*; Gpseu, *G.pseudoferreum*; Gmult, *G.multipileum*; Pfrax, *Perenniporiafraxinea*; Psuba, *P.subacida*. Species and NCBI accession numbers for each of the mitochondrial genomes used are provided in Suppl. material 1: table S1.

The introns are unevenly distributed in the host genes and show a clear preference for *cox1* genes. A total of 374 introns were detected in the mitochondrial genomes of 23 Polyporaceae species, of which 199 were detected in the *cox1* gene, accounting for 53.21% of total introns (Suppl. material 1: table S1). The *cox1* gene is the largest host gene in mitochondrial introns and its intron dynamics could, therefore, significantly affect the organisation and size of mitochondrial genome. Pearson correlation analysis further revealed a high correlation between the number of introns and sizes of mitochondrial genomes of Polyporaceae (Fig. 5A). Introns in Polyporaceae have a significant effect on mitochondrial genome size. Based on the *cox1* gene of *Ganodermacalidophilum*, the introns of the *cox1* gene of 23 Polyporaceae species were divided into different Pcls, while the introns belonging to the same Pcls were considered homologous introns (Fig. 5B). The 199 introns in the *cox1* genes of 23 mitochondrial genomes were classified into 30 Pcl types, indicating the rich diversity of intron types in Polyporaceae. A total of 199 introns were also detected in the *cox1* gene of 23 Polyporaceae species, of which five introns belonged to group II, one was unknown and the rest belonged to group I. A total of 30 Pcls were detected in 23 Polyporaceae species, of which P706 was the most common intron type, detected in 16 Polyporaceae, followed by P612 and P731 in 15 Polyporaceae. Seven of 30 Pcls (S309, P394, P401, P703, P720, P864, P1125) were only detected in one of the 23 Polyporaceae species. Four intron types (P490, P612, P706, P1262) were relatively common, present in all three *Daedaleopsis* species. The differences in intron type and number amongst the three *Daedaleopsis* species further indicated that intron gain/loss occurred during the evolution of the *Daedaleopsis* mitochondrial genomes. Rarer Pcls, such as P900 and P1057, have also been found in *Daedaleopsis*, *Ganoderma* and *Perenniporia*, indicating that there may be potential gene transfer events between the relatively distant species in mitochondrial genome evolution. Compared to *D.sinensis*, the mitochondrial genome of *D.confragosa* contains five non-homologous introns, indicating greater differentiation of intron evolution.

### ﻿Repeats

The repeats of the mitochondrial genome of *D.sinensis* were analysed and 41 SSRs were identified. These included 38 (92.68%) monomeric, one (2.44%) dimeric, one (2.44%) trimeric and one (2.44%) hexameric SSR (Suppl. material 1: tables S4, S5). Overall, seven tandem repeats in the mitochondrial genome of *D.sinensis* were identified, with a similarity of at least 75% and lengths ranging from 6 to 24 bp (Suppl. material 1: table S6). Additionally, 50 pairs of interspersed repeats with a length of at least 30 bp were also discovered, including 30 pairs of forward repeats (F), 19 pairs of palindromic repeats (P) and one pair of complementary repeats (C) (Suppl. material 1: table S7). Suppl. material 2: fig. S2 depicts the distribution of all these repeats in the mitochondrial genome of *D.sinensis*.

### ﻿Rearrangement and homology

**Figure 6.**
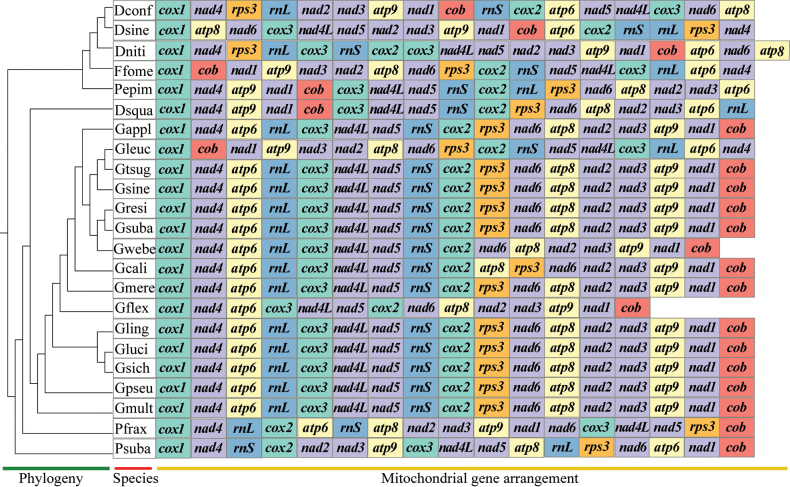
Comparisons of the gene sequences of 23 Polyporaceae species. Starting with *cox1*, all genes, including PCGs and rRNA genes, are displayed in the order in which they occur in the mitochondrial genome.

**Figure 7.**
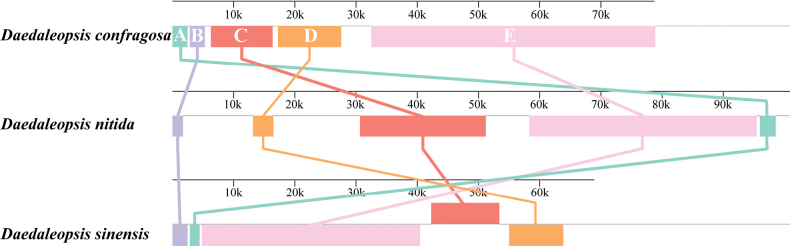
Collinearity analysis of the mitochondrial genes of the three *Daedaleopsis* species. Rectangular blocks of the same colour represent homologous regions between different mitochondrial genomes.

Subsequently, the arrangements of the 15 PCGs and the two rRNA genes were compared in the mitochondrial genomes of the 23 Polyporaceae species (Fig. 6). The comparisons revealed that mitochondrial gene rearrangements were detected between species of different genera. Two gene pairs, *nad2*/*nad3* and *nad4L*/*nad5*, were found in the mitochondrial gene arrangement of 23 Polyporaceae species. However, within the mitochondrial genes of *Fomesfomentarius*, inversion of the *nad2* and *nad3* gene pair was observed. Compared with those of *D.confragosa* and *D.nitida*, the mitochondrial genome of *D.sinensis* presented evidences of large-scale rearrangements, including gene migration, inversion and insertion. Collinearity analysis of the whole mitochondrial genome was further conducted on the three closely-related *Daedaleopsis* species. Five homologous regions (A to E) were detected in each of the mitochondrial genomes (Fig. 7). The homologous region C in the other two species was significantly smaller than that in the mitochondrial genome of *D.nitida*, indicating that the mitochondrial genome contracted during evolution. Collinear analysis of nine phylogenetic relatives revealed further gene rearrangement between *D.sinensis* and related species (Suppl. material 2: fig. S3). *Taiwanofunguscamphoratus*, *Trametescoccinea* and *Wolfiporiacocos* have larger mitochondrial genomes than other species, which may contribute to their greater collinearity. The collinearity results revealed that the mitochondrial genomes of the nine Polyporaceae species were not statistically consistent, indicating that the existence of extensive gene rearrangements occurred between the mitochondrial genomes of *D.sinensis* and its close relatives.

### ﻿Comparative genomics

Comparative analysis of the mitochondrial genomes of 23 Polyporaceae species revealed the significant variation in the size of 23 mitochondrial genomes, ranging from 40,719 to 124,588 bp, with an average size of 79,583 bp (Suppl. material 1: table S1). The GC content of the 23 mitochondrial genomes also varied, ranging from 24.1% to 28.7%, with an average GC content of 26.1%. The three *Daedaleopsis* species contained lower GC content than the average GC content of the 23 mitochondrial genomes. Positive AT skews were found amongst 13 of the 23 mitochondrial genomes, while 20 had positive GC skews. Each of the 23 mitochondrial genomes contains two rRNA genes, while the number of tRNA genes ranged from 22 to 29. The number of introns contained in the 23 mitochondrial genomes also varied widely.

### ﻿Phylogenetics of mitochondrial genomes

**Figure 8.**
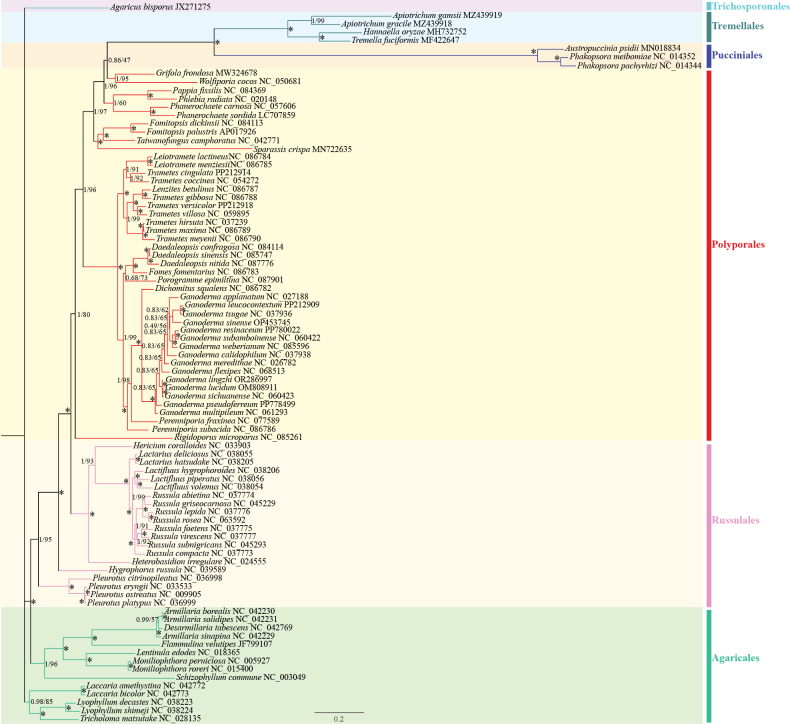
Molecular phylogeny of the 87 investigated species based on BI and ML methods of the conserved mitochondrial PCGs. The BPP and bootstrap values are indicated next to each branch. The asterisks indicate that the BPP and bootstrap values are 1 and 100%, respectively. The GenBank accession numbers are displayed as suffixes following species names.

A phylogenetic tree of 87 fungal species in the Basidiomycota division was constructed using the DNA sequences of conserved PCGs. Suppl. material 1: table S1 lists the species, NCBI accession numbers and characteristics of the mitochondrial genomes investigated in the phylogenetic study. The phylogenetic tree was constructed using the selected common PCGs, which included the *atp6*, *atp8*, *cox1*, *cox2*, *cox3*, *cob*, *nad1*, *nad2*, *nad3*, *nad4*, *nad4L*, *nad5* and *nad6* genes. It also showed that 74 out of the 84 branching nodes had bootstrap values greater than 90%, including 72 nodes with 100% bootstrap values. In comparison, 70 of the 84 branching nodes had Bayesian posterior probability (BPP) values higher than 90, including 54 nodes with 100 BPPs. *D.sinensis* is closely related to the two species within the *Daedaleopsis* genus and is classified on the same branch as *Fomesfomentarius* (Fig. 8).

### ﻿Prediction of RNA editing sites

We identified 43 C-to-U RNA editing sites in 14 mitochondrial PCGs (Suppl. material 1: table S8). Suppl. material 2: fig. S4A presents the distribution of the predicted RNA editing sites in each gene. Amongst these genes, *cox1*, *nad4* and *nad5* had the most RNA editing sites, each with six sites, while *atp9* had no RNA editing sites. This study further revealed that RNA editing events could introduce stop codons in the *nad1* gene.

Suppl. material 2: fig. S4B depicts the effects of RNA editing events on amino acids. These findings indicate that 39 amino acids experienced non-synonymous changes, altering the types of encoded amino acids. The most frequent transition was the Ser to phenylalanine (Phe) transition, followed by the conversion of proline (Pro) to Leu and Ser to Leu. A smaller number of amino acids also underwent synonymous substitution, including isoleucine, Phe, Pro and valine, each with one synonymous substitution. The RNA editing sites involved a total of 18 codon modifications, of which 39.5% resulting in amino acid changes with no change in hydrophobicity, 2.3% were predicted to switch from hydrophobic to hydrophilic and 55.9% changed from hydrophilic to hydrophobic (Suppl. material 1: table S9).

## ﻿Discussion

### ﻿Features of the mitochondrial genome of *Daedaleopsissinensis*

In this study, the mitochondrial genome of *D.sinensis* was sequenced, assembled and analysed. Despite sharing the same genus, the three species all have distinct mitochondrial genome sizes. Fungal mitochondrial genomes range in size from tens to hundreds of kilobases, primarily due to introns, intergenic regions, repeats, horizontal gene transfer and plasmid-derived dynamic change regions (Burger et al. 2003; Wang et al. 2020a, b; Ma et al. 2022). In the Polyporaceae species involved in our study, the largest mitochondrial genome was 3.06 times larger than the smallest. The differences in gene content between the different species indicated that gene gain and loss occur in the mitochondrial genome of fungi during the long-term evolution process, which is very common in the mitochondrial genome. Although the mitochondrial genomes of the three *Daedaleopsis* species differed in size, their PCGs were similar in type and quantity, consisting primarily of *atp6*, *atp8*, *atp9*, *cob*, *cox1*, *cox2*, *cox3*, *nad1*, *nad2*, *nad3*, *nad4*, *nad4L*, *nad5*, *nad6* and *rps3* genes. Variations in gene order amongst different species may account for differences in mitochondrial genome size. In addition, the *dpo* gene found in the mitochondrial genome of *D.sinensis* may be involved in the horizontal gene transfer of DNA polymerase genes to the mitochondrial genome (Férandon et al. 2013). The three *Daedaleopsis* species contain two RNA genes and 26 tRNA genes, but their gene sizes and base composition showed variation. It has been reported that tRNA anticodon arm mutations may also lead to changes in mRNA codon specific recognition and could thus affect protein synthesis (Ding et al. 2019; Lin et al. 2021). As a result, it is necessary to further study the subsequent effects of tRNA mutations.

### ﻿PCGs and repeats

ATG and TAA are the typical start and stop codons used in the mitochondrial genomes. In the present study, codon usage analysis revealed that the third codon position of the mitochondrial genome of *D.sinensis* prefers adenine/thymine (A/T), which is more pronounced and prevalent in higher plants (Yang et al. 2021). The use of codons also plays an important role in gene expression levels and protein structure (Liu et al. 2021). The influence of codon bias on protein structure is reflected primarily in the stage from mRNA translation to protein. The encoding genes of proteins with different tertiary structures often affect the translation extension speed of different regions and regulate the translation efficiency and accuracy by using different codon biases, thereby affecting the folding of new-born peptide chains in the translation process, as well as the spatial conformation of the translated protein (Liu et al. 2021; Moss et al. 2024). Codon preference analysis further helps in understanding the molecular mechanism of gene transcription and translation, determining the optimal codon and predicting some genes with unknown functions (Jiang et al. 2008; Liang 2010).

The evolutionary rate of genes in the mitochondrial genome is generally regulated by purifying selection, mutation and directed selection. The ratio of the non-synonymous replacement rate to the synonymous replacement rate is also an important index to determine the evolutionary selection pressure on PCGs in the mitochondrial genome (Hurst and Jiggins 2005). The gene with the highest *Ka*/*Ks* ratio is the *nad6* gene, while the *Ka* and *Ks* values of the *nad6* gene are close to each other, indicating that the *nad6* gene is primarily subjected to harmless neutral selection during genetic evolution and that it is under weak natural selection pressure. The *Ka*/*Ks* ratio of the *atp6*, *atp9*, *cox1*, *cox3*, *nad1*, *nad4L* and *nad5* genes is relatively low, indicating that these PCGs are under strong natural selection pressure and may be bound by the functions of the proteins encoded by the genes to ensure the normal biological function of the proteins. These findings also indicate that these genes play important roles in the survival and evolution of species. Moreover, the evolution rate of these genes is slow, while the level of genetic variation is low, making them suitable for the study of the phylogenetic evolution of *Daedaleopsis* species, as well as the identification of molecular markers for the construction of biological barcodes between different species. Fungal mitochondrial genomes frequently include SSRs, tandem repeats and interspersed repeats. During evolution of the mitochondrial genome, recombination may have caused these abundant repeats to alter the size of the mitochondrial genome (Burger et al. 2003). The high A/T composition of SSRs may further contribute to the abundance of AT in the mitochondrial genome of *D.sinensis*. The interspersed repeats had a total length of 3,193 bp, accounting for 4.62% (3,193/69,155) of the mitochondrial genome length. The longest palindromic repeat (P) spanned 251 bp, while the longest forward repeat (F) extended to 159 bp. These repeats may also influence the size of the mitochondrial genome, as well as demonstrate the frequency of intermolecular recombination in the mitochondrial genome (Wynn and Christensen 2019).

Repeats are a part of the gene regulatory network, which can co-regulate gene expression with other signalling molecules or homeopathic expression elements and are rich in genetic information (Lu et al. 2020). Some repeats can also specifically bind to certain proteins, thereby facilitating the assembly of nucleic acids into various higher-level structures (Hoskins et al. 2002). As such, repeats greatly expand and enrich genetic information in the process of species evolution and generate the impetus of evolution (Eichler and Sankoff 2003; Lu et al. 2021). Understanding and studying these repeats will contribute to our knowledge of DNA maintenance and evolution in mitochondria (Wynn and Christensen 2019).

### ﻿Intron dynamics

Introns are commonly detected in fungal mitochondrial genomes, while the size and gain/loss of introns may affect the organisation and size of fungal mitochondrial genomes (Sandor et al. 2018). Research has also been shown that introns are the primary factors affecting the size variation of fungal mitochondrial genomes (Friedrich et al. 2012). Even within the single fungal genus, the number and species of introns may vary (Jung et al. 2010; Joardar et al. 2012; Beaudet et al. 2013). Many fungi contain both group I and II introns, but group I introns are more common in fungi than II (Lang et al. 2007). In Basidiomycota, most introns belong to group I, while the *cox1* gene is accepted to be the largest host gene of group I introns (Wang et al. 2020b). Some homologous introns were simultaneously detected in species with relatively distant phylogenetic relationships, which may indicate a potential gene transfer event. Simultaneously, the differences in introns between species in the same family or genus may indicate the variability and dynamics of intron distribution amongst species (Li et al. 2022). Further sequencing and analysis will be needed to reveal the origin of these introns, including transfer events and evolutionary mechanism.

### ﻿RNA editing sites

The 43 RNA editing sites identified were distributed across 14 PCGs in the mitochondrial genome of *D.sinensis*. RNA editing events cause amino acid composition variations, resulting in differences in encoded information. RNA editing is essential for the synthesis of functional proteins in the mitochondrial system. These functional proteins exhibit closer sequence conservation after editing with homologues in other systems (Brennicke et al. 1999). Further, RNA editing events in *D.sinensis* were predicted. RNA editing may also alter the codon initiation and termination of PCGs. However, the initiation and termination codons triggered by RNA editing may result in more conserved proteins, thereby regulating mitochondrial gene expression (Galtier 2011). RNA editing also altered 55.9% of the amino acids in *D.sinensis* from hydrophilic to hydrophobic. This change in hydrophobicity has previously been shown to be closely related to protein conformation and function (Brenner et al. 2019). The hydrophobicity of amino acids reflects protein folding, while their hydrophilic/hydrophobic interactions are amongst the most important forces for maintaining protein tertiary structure (Lacey et al. 1992). The prediction of RNA editing events facilitates the study of mitochondrial gene expression mechanisms and is a valuable reference to predict gene function (Li et al. 2014; Hao et al. 2021).

### ﻿Collinearity and gene rearrangement

Homology analysis is another analytical approach crucial for elucidating species evolution (Bonierbale et al. 1988; Tanksley et al. 1988; Lagercrantz et al. 1996; Wang et al. 2015). The present study explored the homologous collinear regions between *D.sinensis* and other eight fungi, while the more heterogeneous collinear maps revealed the presence of gene rearrangements in the mitochondrial genome of *D.sinensis*, which could function as potential drivers of *D.sinensis* evolution (Yang et al. 2016; Ye et al. 2018). Mitochondrial genome rearrangement is common in plants, animals and fungi, while the analysis of variations in mitochondrial gene sequences can reveal the phylogenetic status of a species and its phylogenetic relationships (Sankoff et al. 1992; Boore 1999; Li et al. 2018, 2019a). Our study revealed widespread gene inversion, insertion and migration amongst the three *Daedaleopsis* species, indicating that mitochondrial gene organisation is highly variable. Herein, several repeats were simultaneously discovered in the mitochondrial genome of *D.sinensis*. According to previous studies, the accumulation of repeats is one of the primary causes of fungal mitochondrial gene rearrangement (Li et al. 2020; Chen et al. 2021). Simultaneously, the arrangement of mitochondrial genes can provide important information regarding genetic variation and phylogenetic relationships between species (Zheng et al. 2018; Li et al. 2021). A large number of studies have shown that a more complex rearrangement mechanism may exist in fungal mitochondrial genomes than in animals (Song et al. 2024). The gene arrangement of 23 species in the family Polyporaceae also shows its complexity.

### ﻿Phylogenetics of mitochondrial genomes

Herein, a phylogenetic tree involving 87 Basidiomycota species was constructed using both BI and ML analyses of the concatenated conserved mitochondrial PCG genes (Fig. 8). Four species, namely, *D.confragosa*, *D.sinensis*, *D.nitida* and *F.fomentarius*, were grouped in a closely-related cluster with high support values. Moreover, *D.sinensis* shares a tighter evolutionary link with *D.confragosa* than other relatives. Compared with phylogenetic trees constructed from whole genomes, phylogenetic trees constructed from mitochondrial PCGs have certain similarities (Ma et al. 2024). However, the mitochondrial phylogenetic tree had a relatively low support for several other branches. Hence, the evolutionary lineages on the basis of mitochondrial genomes may not adequately reflect actual evolutionary links. Mitochondria, which are semi-autonomous organelles with their own genetic expression system, may evolve in divergent directions from the nuclear genome (Yin et al. 2015; Liu et al. 2020, 2022). Mitochondrial DNA, which evolves at a faster rate than nuclear DNA, is easy to analyse and can be used to assess the interrelationships between different genera or species of fungi and between different strains of the same species (Ye et al. 2018).

## ﻿Conclusions

This study is the first to assemble the mitochondrial genome of *D.sinensis* using the second- and third-generation sequencing technologies. Herein, the mitochondrial genome of *D.sinensis* was successfully assembled and annotated, which is 69,155 bp long and has a GC content of 25.0%. Additionally, 44 genes were annotated, including 15 PCGs, 26 tRNA genes, two rRNA genes and one *dpo* gene. Further, a comprehensive analysis of the codon preference, variation and evolution of PCGs, RNA genes, repeats, gene rearrangements, intron dynamics, RNA editing events and phylogenetic relationships of *D.sinensis* and several related species was conducted to clarify mitochondrial genomic features and expand the fungal mitochondrial genomic database. This improves our understanding of the genetic characteristics and developmental relationships of mitochondrial organelles in fungi. These findings will be valuable references for further research on fungal evolution and ecological functions.

## ﻿Additional information

### Author ORCIDs

Jin-Xin Ma https://orcid.org/0000-0003-2069-7493

Hai-Jiao Li https://orcid.org/0000-0002-7227-4479

Can Jin https://orcid.org/0000-0003-4573-6911

Hao Wang https://orcid.org/0000-0002-4880-7001

Lu-Xin Tang https://orcid.org/0000-0002-5753-6354

Jing Si https://orcid.org/0000-0001-9229-0727

Bao-Kai Cui https://orcid.org/0000-0003-3059-9344

### Data availability

The complete mitochondrial genome of *D.sinensis* has been deposited in the GenBank database under the accession number NC_085747. All data generated or analysed during this study are included in this article [and its Suppl. materials 1, 2].

## Supplementary materials

Supplementary material 1 Supplementary tables Data type xlsx Explanation note **table S1**. Species information and GenBank accession numbers used for the phylogenetic analysis in this study. **table S2**. Protein information encoded by the mitochondrial genome of *Daedaleopsissinensis*. **table S3**. RSCU of the individual amino acid pairs of codons in the mitochondrial genome of *Daedaleopsissinensis*. **table S4**. SSRs in the mitochondrial genome of *Daedaleopsissinensis*. **table S5**. Data analysis of SSRs in the mitochondrial genome of *Daedaleopsissinensis*. **table S6**. Tandem repeats in the mitochondrial genome of *Daedaleopsissinensis*. **table S7**. Interspersed repeats in the mitochondrial genome of *Daedaleopsissinensis*. **table S8**. Potential RNA editing sites in PCGs of the mitochondrial genome of *Daedaleopsissinensis*. **table S9**. Amino acid changes in the potential RNA editing sites of PCGs in the mitochondrial genome of *Daedaleopsissinensis*. https://binary.pensoft.net/file/1253220This dataset is made available under the Open Database License (http://opendatacommons.org/licenses/odbl/1.0/). The Open Database License (ODbL) is a license agreement intended to allow users to freely share, modify, and use this Dataset while maintaining this same freedom for others, provided that the original source and author(s) are credited.Jin-Xin Ma, Hai-Jiao Li, Can Jin, Hao Wang, Lu-Xin Tang, Jing Si, Bao-Kai Cui

Supplementary material 2 Supplementary images Data type docx Explanation note **figure S1**. Putative secondary structures of tRNA genes identified in the mitochondrial genomes of *Daedaleopsissinensis*. The tRNAs in green or red fonts represent tRNAs shared by the three *Daedaleopsis* species, while the tRNA in blue font represents tRNA found only in *D.nitida*. Residues conserved across the three mitochondrial genomes are shown in green, while variable sites are shown in red. All genes are shown in their orders of occurrence in the mitochondrial genome of *D.sinensis* starting from *trnS*. **figure S2**. Distribution of repeats in the mitochondrial genome of *Daedaleopsissinensis*. The outermost circle represents the SSRs, followed by the tandem repeats, while the lines in the innermost circle represent interspersed repeats. **figure S3**. Collinearity analysis amongst the nine mitochondrial genomes. Bars represent the mitochondrial genomes, while ribbons indicate the homologous sequences between adjacent species. **figure S4**. Characteristics of the RNA editing sites identified in the mitochondrial PCGs of *Daedaleopsissinensis*. **A** Diagram of the distribution of RNA editing sites in the mitochondrial PCGs of *D.sinensis*. Bars depict the number of RNA editing sites for each gene; **B** Statistics on the potential effects of RNA editing events. The x-axis represents the changes in amino acids, while the y-axis represents the number of times each change occurred. https://binary.pensoft.net/file/1253218This dataset is made available under the Open Database License (http://opendatacommons.org/licenses/odbl/1.0/). The Open Database License (ODbL) is a license agreement intended to allow users to freely share, modify, and use this Dataset while maintaining this same freedom for others, provided that the original source and author(s) are credited.Jin-Xin Ma, Hai-Jiao Li, Can Jin, Hao Wang, Lu-Xin Tang, Jing Si, Bao-Kai Cui

## References

[B1] Aguileta G de Vienne DM Ross ON Hood ME Giraud T Petit E Gabaldón T (2014) High variability of mitochondrial gene order among fungi. Genome Biology and Evolution 6(2): 451–465. 10.1093/gbe/evu028 24504088 PMC3942027

[B2] Basse CW (2010) Mitochondrial inheritance in fungi. Current Opinion in Microbiology 13(6): 712–719. 10.1016/j.mib.2010.09.003 20884279

[B3] Beaudet D Nadimi M Iffis B Hijri M (2013) Rapid mitochondrial genome evolution through invasion of mobile elements in two closely related species of arbuscular mycorrhizal fungi. PLoS ONE 8(4): 12. 10.1371/journal.pone.0060768 PMC3630166 23637766

[B4] Beier S Thiel T Münch T Scholz U Mascher M (2017) MISA-web: a web server for microsatellite prediction. Bioinformatics 33(16): 2583–2585. 10.1093/bioinformatics/btx198 28398459 PMC5870701

[B5] Benson G (1999) Tandem repeats finder: a program to analyze DNA sequences. Nucleic Acids Research 27(2): 573–580. 10.1093/nar/27.2.573 9862982 PMC148217

[B6] Bonierbale MW Plaisted RL Tanksley SD (1988) RFLP maps based on a common set of clones reveal modes of chromosomal evolution in potato and tomato. Genetics 120(4): 1095–1103. 10.1093/genetics/120.4.1095 17246486 PMC1203572

[B7] Boore JL (1999) Animal mitochondrial genomes. Nucleic Acids Research 27: 1767–1780. 10.1093/nar/27.8.1767 10101183 PMC148383

[B8] Brenner WG Mader M Müller NA Hoenicka H Schroeder H Zorn I Fladung M Kersten B (2019) High level of conservation of mitochondrial RNA editing sites among four *Populus* species. G3: Genes, Genomes, Genetics 9(3): 709–717. 10.1534/g3.118.200763 PMC6404595 30617214

[B9] Brennicke A Marchfelder A Binder S (1999) RNA editing. FEMS Microbiology Reviews 23(3): 297–316. 10.1111/j.1574-6976.1999.tb00401.x 10371035

[B10] Burger G Gray MW Lang BF (2003) Mitochondrial genomes: anything goes. Trend in Genetics 19(12): 709–716. 10.1016/j.tig.2003.10.012 14642752

[B11] Chan PP Lowe TM (2019) tRNAscan-SE: searching for tRNA genes in genomic sequences. In: Kollmar M (Ed.) Gene prediction.Methods in Molecular Biology, Humana, New York, 1–14. 10.1007/978-1-4939-9173-0_1 PMC6768409 31020551

[B12] Chatre L Ricchetti M (2014) Are mitochondria the Achilles’ heel of the Kingdom Fungi? Current Opinion in Microbiology 20: 49–54. 10.1016/j.mib.2014.05.001 24906191

[B13] Chen C Li Q Fu RT Wang J Deng GM Chen XJ Lu DH (2021) Comparative mitochondrial genome analysis reveals intron dynamics and gene rearrangements in two *Trametes* species. Scientific Reports 11(1): 2569. 10.1038/s41598-021-82040-7 33510299 PMC7843977

[B14] Chen Y Ye WC Zhang YD Xu YS (2015) High speed BLASTN: an accelerated MegaBLAST search tool. Nucleic Acids Research 43(16): 7762–7768. 10.1093/nar/gkv784 26250111 PMC4652774

[B15] Dai YC (1996) Changbai wood-rotting fungi 7. A checklist of the polypores. Fungal Science 11: 79–105.

[B16] Darling ACE Mau B Blattner FR Perna NT (2004) Mauve: multiple alignment of conserved genomic sequence with rearrangements. Genome Research 14(7): 1394–1403. 10.1101/gr.2289704 15231754 PMC442156

[B17] Ding Y Teng YS Zhuo GC Xia BH Leng JH (2019) The mitochondrial tRNA^His^ G12192A mutation may modulate the clinical expression of deafness-associated tRNA^Thr^ G15927A mutation in a Chinese pedigree. Current Molecular Medicine 19: 136–146. 10.2174/1566524019666190308121552 30854964

[B18] Donath A Jühling F Al-Arab M Bernhart SH Reinhardt F Stadler PF Middendorf M Bernt M (2019) Improved annotation of protein-coding genes boundaries in metazoan mitochondrial genomes. Nucleic Acids Research 47(20): 10543–10552. 10.1093/nar/gkz833 31584075 PMC6847864

[B19] Edera AA Small I Milone DH Sanchez-Puerta MV (2021) Deepred-Mt: deep representation learning for predicting C-to-U RNA editing in plant mitochondria. Computers in Biology and Medicine 136: 104682. 10.1016/j.compbiomed.2021.104682 34343887

[B20] Eichler EE Sankoff D (2003) Structural dynamics of eukaryotic chromosome evolution. Science 301(5634): 793–797. 10.1126/science.1086132 12907789

[B21] Férandon C Moukha S Callac P Benedetto JP Castroviejo M Barroso G (2010) The *Agaricusbisporus cox1* gene: the longest mitochondrial gene and the largest reservoir of mitochondrial group I introns. PLoS ONE 5(11): e14048. 10.1371/journal.pone.0014048 PMC2987802 21124976

[B22] Férandon C Xu JP Barroso G (2013) The 135 kbp mitochondrial genome of *Agaricusbisporus* is the largest known eukaryotic reservoir of group I introns and plasmid-related sequences. Fungal Genetics and Biology 55: 85–91. 10.1016/j.fgb.2013.01.009 23428625

[B23] Friedrich A Jung PP Hou J Neuvéglise C Schacherer J (2012) Comparative mitochondrial genomics within and among yeast species of the *Lachancea* genus. PLoS ONE 7(10): 6. 10.1371/journal.pone.0047834 PMC3480396 23112855

[B24] Galtier N (2011) The intriguing evolutionary dynamics of plant mitochondrial DNA. BMC Biology 9: 61. 10.1186/1741-7007-9-61 PMC3181201 21951676

[B25] Greiner S Lehwark P Bock R (2019) OrganellarGenomeDRAW (OGDRAW) version 1.3.1: expanded toolkit for the graphical visualization of organellar genomes. Nucleic Acids Research 47: W59–W64. 10.1093/nar/gkz238 PMC6602502 30949694

[B26] Han XH He H Shen HY Tang JH Dong WY Shi YF Wu SQ Zhang FP Liang GH (2020) Comparative mitochondrial genome analysis of *Dendrolimushoui* (Lepidoptera: Lasiocampidae) and phylogenetic relationship among Lasiocampidae species. PLoS ONE 15(5): e0232527. 10.1371/journal.pone.0232527 PMC7224488 32407393

[B27] Hao W Liu GX Wang WP Shen W Zhao YP Sun JL Yang QY Zhang YX Fan WJ Pei SS Chen ZQ Xu DB Qin TF (2021) RNA editing and its roles in plant organelles. Frontiers in Genetics 12: 757109. 10.3389/fgene.2021.757109 PMC8511385 34659369

[B28] Hoskins RA Smith CD Carlson JW Carvalho AB Halpern A Kaminker JS Kennedy C Mungall CJ Sullivan BA Sutton GG Yasuhara JC Wakimoto BT Myers EW Celniker SE Rubin GM Karpen GH (2002) Heterochromatic sequences in a *Drosophila* whole-genome shotgun assembly. Genome Biology 3(12): RESEARCH0085. 10.1186/gb-2002-3-12-research0085 PMC151187 12537574

[B29] Hurst GDD Jiggins FM (2005) Problems with mitochondrial DNA as a marker in population, phylogeographic and phylogenetic studies: the effects of inherited symbionts. Proceedings of the Royal Society B, Biological Sciences 272(1572): 1525–1534. 10.1098/rspb.2005.3056 PMC1559843 16048766

[B30] Jiang Y Deng F Wang HL Hu ZH (2008) An extensive analysis on the global codon usage pattern of baculoviruses. Archives of Virology 153(12): 2273–2282. 10.1007/s00705-008-0260-1 19030954

[B31] Jin JJ Yu WB Yang JB Song Y dePamphilis CW Yi TS Li DZ (2020) GetOrganelle: a fast and versatile toolkit for accurate *de novo* assembly of organelle genomes. Genome Biology 21(1): 241. 10.1186/s13059-020-02154-5 32912315 PMC7488116

[B32] Joardar V Abrams NF Hostetler J Paukstelis PJ Pakala S Pakala SB Zafar N Abolude OO Payne G Andrianopoulos A Denning DW Nierman WC (2012) Sequencing of mitochondrial genomes of nine *Aspergillus* and *Penicillium* species identifies mobile introns and accessory genes as main sources of genome size variability. BMC Genomics 13: 698. 10.1186/1471-2164-13-698 PMC3562157 23234273

[B33] Jung PP Friedrich A Souciet JL Louis V Potier S de Montigny J Schacherer J (2010) Complete mitochondrial genome sequence of the yeast *Pichiafarinosa* and comparative analysis of closely related species. Current Genetics 56(6): 507–515. 10.1007/s00294-010-0318-y 20830585

[B34] Katoh K Standley DM (2013) MAFFT multiple sequence alignment software version 7: improvements in performance and usability. Molecular Biology and Evolution 30(4): 772–780. 10.1093/molbev/mst010 23329690 PMC3603318

[B35] Krzywinski M Schein J Birol I Connors J Gascoyne R Horsman D Jones SJ Marra MA (2009) Circos: an information aesthetic for comparative genomics. Genome Research 19(9): 1639–1645. 10.1101/gr.092759.109 19541911 PMC2752132

[B36] Kurtz S Choudhuri JV Ohlebusch E Schleiermacher C Stoye J Giegerich R (2001) REPuter: the manifold applications of repeat analysis on a genomic scale. Nucleic Acids Research 29(22): 4633–4642. 10.1093/nar/29.22.4633 11713313 PMC92531

[B37] Lacey Jr JC Wickramasinghe NSMD Sabatini RS (1992) Preferential hydrophobic interactions are responsible for a preference of D-amino acids in the aminoacylation of 5’-AMP with hydrophobic amino acids. Experientia 48(4): 379–383. 10.1007/BF01923434 1582495

[B38] Lagercrantz U Putterill J Coupland G Lydiate D (1996) Comparative mapping in *Arabidopsis* and *Brassica*, fine scale genome collinearity and congruence of genes controlling flowering time. The Plant Journal 9(1): 13–20. 10.1046/j.1365-313X.1996.09010013.x 8580970

[B39] Lanfear R Frandsen PB Wright AM Senfeld T Calcott B (2017) PartitionFinder 2: new methods for selecting partitioned models of evolution for molecular and morpho­logical phylogenetic analyses. Molecular Biology and Evolution 34(3): 772–773. 10.1093/molbev/msw260 28013191

[B40] Lang BF Laforest MJ Burger G (2007) Mitochondrial introns: a critical view. Trend in Genetics 23(3): 119–125. 10.1016/j.tig.2007.01.006 17280737

[B41] Larsen MJ Jurgenson MF Harvey AE (1979) N_2_ fixation associated with wood decayed by some common fungi in western Montana. Canadian Journal of Forest Research 8(3): 341–345. 10.1139/x78-050

[B42] Larsen MJ Jurgenson MF Harvey AE (1982) N_2_ fixation in brown-rotted soil wood in an intermountain cedar-hemlock ecosystem. Forest Science 28(2): 292–296.

[B43] Letunic I Bork P (2007) Interactive Tree Of Life (iTOL): an online tool for phylogenetic tree display and annotation. Bioinformatics 23(1): 127–128. 10.1093/bioinformatics/btl529 17050570

[B44] Li HJ Si J He SH (2016) *Daedaleopsishainanensis* sp. nov. (Polyporaceae, Basidiomycota) from tropical China based on morphological and molecular evidence. Phytotaxa 275(3): 294–300. 10.11646/phytotaxa.275.3.7

[B45] Li Q Bao ZJ Tang K Feng HY Tu WY Li LJ Han YL Cao M Zhao CS (2022) First two mitochondrial genomes for the order *Filobasidiales* reveal novel gene rearrangements and intron dynamics of *Tremellomycetes*. IMA Fungus 13(1): 7. 10.1186/s43008-022-00094-2 35501936 PMC9059411

[B46] Li Q Chen C Xiong C Jin X Chen ZQ Huang WL (2018) Comparative mitogenomics reveals large-scale gene rearrangements in the mitochondrial genome of two *Pleurotus* species. Applied Microbiology and Biotechnology 102: 6143–6153. 10.1007/s00253-018-9082-6 29799088

[B47] Li Q Li LJ Feng HY Tu WY Bao ZJ Xiong C Wang X Qing Y Huang WL (2021) Characterization of the complete mitochondrial genome of basidiomycete yeast *Hannaellaoryzae*: intron evolution, gene rearrangement, and its phylogeny. Frontiers in Microbiology 12: 646567. 10.3389/fmicb.2021.646567 PMC8193148 34122362

[B48] Li Q Ren YH Shi XD Peng LX Zhao JL Song Y Zhao G (2019a) Comparative mitochondrial genome analysis of two ectomycorrhizal fungi (*Rhizopogon*) reveals dynamic changes of intron and phylogenetic relationships of the subphylum Agaricomycotina. International Journal of Molecular Sciences 20(20): 5167. 10.3390/ijms20205167 31635252 PMC6829451

[B49] Li Q Ren YH Xiang DB Shi XD Zhao JL Peng LX Zhao G (2020) Comparative mitogenome analysis of two ectomycorrhizal fungi (*Paxillus*) reveals gene rearrangement, intron dynamics, and phylogeny of basidiomycetes. IMA Fungus 11: 12. 10.1186/s43008-020-00038-8 PMC7333402 32670777

[B50] Li Q Xiang DB Wan Y Wu Q Wu XY Ma CR Song Y Zhao G Huang WL (2019b) The complete mitochondrial genomes of five important medicinal *Ganoderma* species: features, evolution, and phylogeny. International Journal of Biological Macromolecules 139: 397–408. 10.1016/j.ijbiomac.2019.08.003 31381907

[B51] Li XJ Zhang YF Hou MM Sun F Shen Y Xiu ZH Wang XM Chen ZL Sun SSM Small I Tan BC (2014) *Small kernel 1* encodes a pentatricopeptide repeat protein required for mitochondrial *nad7* transcript editing and seed development in maize (*Zeamays*) and rice (*Oryzasativa*). The Plant Journal 79(5): 797–809. 10.1111/tpj.12584 24923534

[B52] Liang FF (2010) Influencing factors of codon bias and its research significance. Animal Husbandry and Feed Science 31(1): 118–119.

[B53] Lin Y Xu XB Wang W Liu FC Zhao DD Li DL Ji KQ Li W Zhao YY Yan CZ (2021) A mitochondrial myopathy-associated tRNA^Ser(UCN)^ 7453G>A mutation alters tRNA metabolism and mitochondrial function. Mitochondrion 57: 1–8. 10.1016/j.mito.2020.11.015 33279600

[B54] Liu BB Ren C Kwak M Hodel R Xu C He J Zhou WB Huang CH Ma H Qian GZ Hong DY Wen J (2022) Phylogenomic conflict analyses in the apple genus *Malus* s.l. reveal widespread hybridization and allopolyploidy driving diversification, with insights into the complex biogeographic history in the Northern Hemisphere. Journal of Integrative Plant Biology 64(5): 1020–1043. 10.1111/jipb.13246 35274452

[B55] Liu LX Du YX Folk RA Wang SY Soltis DE Shang FD Li P (2020) Plastome evolution in Saxifragaceae and multiple plastid capture events involving *Heuchera* and *Tiarella*. Frontiers in Plant Science 11: 361. 10.3389/fpls.2020.00361 PMC7193090 32391025

[B56] Liu Y Yang Q Zhao FZ (2021) Synonymous but not silent: the codon usage code for gene expression and protein folding. Annual Review of Biochemistry 90: 375–401. 10.1146/annurev-biochem-071320-112701 PMC8284178 33441035

[B57] Lloyd CG (1922) Mycological notes 66. Mycological writings 7(66): 1105–1136.

[B58] Lu JYY Chang L Li T Wang T Yin YF Zhan G Han X Zhang K Tao YB Percharde M Wang L Peng Q Yan PX Zhang H Bi XJ Shao W Hong YT Wu ZY Ma RZ Wang PZ Li WZ Zhang J Chang Z Hou YP Zhu B Ramalho-Santos M Li PL Xie W Na J Sun YJ Shen XH (2021) Homotypic clustering of L1 and B1/Alu repeats compartmentalizes the 3D genome. Cell Research 31(6): 613–630. 10.1038/s41422-020-00466-6 33514913 PMC8169921

[B59] Lu JYY Shao W Chang L Yin YF Li T Zhang H Hong YT Percharde M Guo LR Wu ZY Liu LC Liu W Yan PX Ramalho-Santos M Sun YJ Shen XH (2020) Genomic repeats categorize genes with distinct functions for orchestrated regulation. Cell Reports 30(10): 3296–3311. 10.1016/j.celrep.2020.02.048 32160538 PMC7195444

[B60] Ma JX Wang H Jin C Ye YF Tang LX Si J Song J (2024) Whole genome sequencing and annotation of *Daedaleopsissinensis*, a wood-decaying fungus significantly degrading lignocellulose. Frontiers in Bioengineering and Biotechnology 11: 1325088. 10.3389/fbioe.2023.1325088 PMC10826855 38292304

[B61] Ma QZ Geng YH Li Q Cheng CY Zang R Guo YS Wu HY Xu C Zhang M (2022) Comparative mitochondrial genome analyses reveal conserved gene arrangement but massive expansion/contraction in two closely related *Exserohilum* pathogens. Computational and Structural Biotechnology Journal 20: 1456–1469. 10.1016/j.csbj.2022.03.016 35386100 PMC8956966

[B62] Mardanov AV Beletsky AV Kadnikov VV Ignatov AN Ravin NV (2014) The 203 kbp mitochondrial genome of the phytopathogenic fungus *Sclerotiniaborealis* reveals multiple invasions of introns and genomic duplications. PLoS ONE 9: e107536. 10.1371/journal.pone.0107536 PMC4162613 25216190

[B63] Miao YJ Chen HM Xu WQ Liu C Huang LF (2022) *Cistanche* species mitogenomes suggest diversity and complexity in *Lamiales*-order mitogenomes. Genes 13(10): 1791. 10.3390/genes13101791 36292676 PMC9602076

[B64] Moss MJ Chamness LM Clark PL (2024) The effects of codon usage on protein structure and folding. Annual Review of Biophysics 53(1): 87–108. 10.1146/annurev-biophys-030722-020555 PMC11227313 38134335

[B65] Nguyen LT Schmidt HA von Haeseler A Minh BQ (2015) IQ-TREE: a fast and effective stochastic algorithm for estimating maximum-likelihood phylogenies. Molecular Biology and Evolution 32(1): 268–274. 10.1093/molbev/msu300 25371430 PMC4271533

[B66] Núñez M Ryvarden L (2001) East Asian Polypores 2. Polyporaceae s. lato. Synop Fungorum 14: 170–522.

[B67] Osiewacz HD (2002) Aging in fungi: role of mitochondria in *Podosporaanserina*. Mechanisms of Ageing and Development 123(7): 755–764. 10.1016/S0047-6374(01)00421-3 11869733

[B68] Paquin B Laforest MJ Forget L Roewer I Wang Z Longcore J Lang BF (1997) The fungal mitochondrial genome project: evolution of fungal mitochondrial genomes and their gene expression. Current Genetics 31(5): 380–395. 10.1007/s002940050220 9162109

[B69] Ranwez V Douzery EJP Cambon C Chantret N Delsuc F (2018) MACSE v2: toolkit for the alignment of coding sequences accounting for frameshifts and stop codons. Molecular Biology and Evolution 35: 2582–2584. 10.1093/molbev/msy159 30165589 PMC6188553

[B70] Ronquist F Teslenko M van der Mark P Ayres DL Darling A Höhna S Larget B Liu L Suchard MA Huelsenbeck JP (2012) MrBayes 3.2: efficient Bayesian phylogenetic inference and model choice across a large model space. Systematic Biology 61: 539–542. 10.1093/sysbio/sys029 22357727 PMC3329765

[B71] Rozewicki J Li SL Amada KM Standley DM Katoh K (2019) MAFFT-DASH: integrated protein sequence and structural alignment. Nucleic Acids Research 47(W1): W5–W10. 10.1093/nar/gkz342 PMC6602451 31062021

[B72] Sandor S Zhang YJ Xu JP (2018) Fungal mitochondrial genomes and genetic polymorphisms. Applied Microbiology and Biotechnology 102(22): 9433–9448. 10.1007/s00253-018-9350-5 30209549

[B73] Sankoff D Leduc G Antoine N Paquin B Lang BF Cedergren R (1992) Gene order comparisons for phylogenetic inference: evolution of the mitochondrial genome. Proceedings of the National Academy of Sciences of the United States of America 89(14): 6575–6579. 10.1073/pnas.89.14.6575 1631158 PMC49544

[B74] Sharp PM Li WH (1986) Codon usage in regulatory genes in *Escherichiacoli* does not reflect selection for ‘rare’ codons. Nucleic Acids Research 14(19): 7737–7749. 10.1093/nar/14.19.7737 3534792 PMC311793

[B75] Song N Geng YH Li XH (2020) The mitochondrial genome of the phytopathogenic fungus *Bipolarissorokiniana* and the utility of mitochondrial genome to infer phylogeny of Dothideomycetes. Frontiers in Microbiology 11: 863. 10.3389/fmicb.2020.00863 PMC7225605 32457727

[B76] Song XZ Geng YH Xu C Li JX Guo YS Shi Y Ma QZ Li Q Zhang M (2024) The complete mitochondrial genomes of five critical phytopathogenic *Bipolaris* species: features, evolution, and phylogeny. IMA Fungus 15: 15. 10.1186/s43008-024-00149-6 PMC11167856 38863028

[B77] Talavera G Castresana J (2007) Improvement of phylogenies after removing divergent and ambiguously aligned blocks from protein sequence alignments. Systematic Bio­logy 56: 564–577. 10.1080/10635150701472164 17654362

[B78] Tamura K Stecher G Kumar S (2021) MEGA11: molecular evolutionary genetics analysis version 11. Molecular Biology and Evolution 38(7): 3022–3027. 10.1093/molbev/msab120 33892491 PMC8233496

[B79] Tanksley SD Bernatzky R Lapitan NL Prince JP (1988) Conservation of gene repertoire but not gene order in pepper and tomato. Proceedings of the National Academy of Sciences of the United States of America 85(17): 6419–6423. 10.1073/pnas.85.17.6419 16593975 PMC281983

[B80] Thiel T Michalek W Varshney R Graner A (2003) Exploiting EST databases for the development and characterization of gene-derived SSR-markers in barley (*Hordeumvulgare* L.). Theoretical and Applied Genetics 106(3): 411–422. 10.1007/s00122-002-1031-0 12589540

[B81] Thompson JD Higgins DG Gibson TJ (1994) CLUSTAL W: improving the sensitivity of progressive multiple sequence alignment through sequence weighting, position-specific gap penalties and weight matrix choice. Nucleic Acids Research 22(22): 4673–4680. 10.1093/nar/22.22.4673 7984417 PMC308517

[B82] Wang DP Zhang YB Zhang Z Zhu J Yu J (2010) KaKs_Calculator 2.0: a toolkit incorporating gamma-series methods and sliding window strategies. Genomics, Proteomics & Bioinformatics 8(1): 77–80. 10.1016/S1672-0229(10)60008-3 PMC5054116 20451164

[B83] Wang X Jia LH Wang MD Yang H Chen MY Li X Liu HY Li Q Liu N (2020a) The complete mitochondrial genome of medicinal fungus *Taiwanofunguscamphoratus* reveals gene rearrangements and intron dynamics of *Polyporales*. Scientific Reports 10(1): 16500. 10.1038/s41598-020-73461-x 33020532 PMC7536210

[B84] Wang XY Wang JP Jin DC Guo H Lee TH Liu T Paterson AH (2015) Genome alignment spanning major Poaceae lineages reveals heterogeneous evolutionary rates and alters inferred dates for key evolutionary events. Molecular Plant 8(6): 885–898. 10.1016/j.molp.2015.04.004 25896453

[B85] Wang X Wang YJ Yao W Shen JW Chen MY Gao M Ren JN Li Q Liu N (2020b) The 256 kb mitochondrial genome of *Clavariafumosa* is the largest among phylum Basidiomycota and is rich in introns and intronic ORFs. IMA Fungus 11(1): 26. 10.1186/s43008-020-00047-7 33292749 PMC7666478

[B86] Wang YP Tang HB Debarry JD Tan X Li JP Wang XY Lee TH Jin HZ Marler B Guo H Kissinger JC Paterson AH (2012) MCScanX: a toolkit for detection and evolutionary analysis of gene synteny and collinearity. Nucleic Acids Research 40(7): e49. 10.1093/nar/gkr1293 PMC3326336 22217600

[B87] Watanabe M Lee K Goto K Kumagai S Sugita-Konishi Y Hara-Kudo Y (2010) Rapid and effective DNA extraction method with bead grinding for a large amount of fungal DNA. Journal of Food Protection 73(6): 1077–1084. 10.4315/0362-028X-73.6.1077 20537263

[B88] Wick RR Schultz MB Zobel J Holt KE (2015) Bandage: interactive visualization of *de novo* genome assemblies. Bioinformatics 31(20): 3350–3352. 10.1093/bioinformatics/btv383 26099265 PMC4595904

[B89] Wynn EL Christensen AC (2019) Repeats of unusual size in plant mitochondrial genomes: identification, incidence and evolution. G3: Genes, Genomes, Genetics 9(2): 549–559. 10.1534/g3.118.200948 PMC6385970 30563833

[B90] Yang HX Li WH Yu XL Zhang XY Zhang ZY Liu YX Wang WX Tian XX (2021) Insights into molecular structure, genome evolution and phylogenetic implication through mitochondrial genome sequence of *Gleditsiasinensis*. Scientific Reports 11(1): 14850. 10.1038/s41598-021-93480-6 34290263 PMC8295344

[B91] Yang J Liu G Zhao N Chen S Liu D Ma W Hu Z Zhang M (2016) Comparative mitochondrial genome analysis reveals the evolutionary rearrangement mechanism in *Brassica*. Plant Biology 18(3): 527–536. 10.1111/plb.12414 27079962

[B92] Ye LY Meng GL Cheng B Zhao LL Xie F Wu XP (2018) Mitochondrial genome sequencing and polymorphism analysis of *Pleurotuspulmonarius*. Mycosystema 37(9): 1179–1187. 10.13346/j.mycosystema.180081

[B93] Yin H Akimoto M Kaewcheenchai R Sotowa M Ishii T Ishikawa R (2015) Inconsistent diversities between nuclear and plastid genomes of AA genome species in the genus *Oryza*. Genes Genet Syst 90(5): 269–281. 10.1266/ggs.14-00063 26687860

[B94] Zhang D Gao FL Jakovlić I Zou H Zhang J Li WX Wang GT (2020) PhyloSuite: an integrated and scalable desktop platform for streamlined molecular sequence data management and evolutionary phylogenetics studies. Molecular Ecology Resources 20(1): 348–355. 10.1111/1755-0998.13096 31599058

[B95] Zheng BY Cao LJ Tang P van Achterberg K Hofmann AA Chen HY Chen XX Wei SJ (2018) Gene arrangement and sequence of mitochondrial genomes yield insights into the phylogeny and evolution of bees and sphecid wasps (Hymenoptera: Apoidea). Molecular Phylogenetics and Evolution 124: 1–9. 10.1016/j.ympev.2018.02.028 29510236

