# Supplementary material for: ﻿Assembly and comparative analysis of the complete mitochondrial genome of Daedaleopsissinensis (Polyporaceae, Basidiomycota), contributing to understanding fungal evolution and ecological functions
Source: IMA Fungus. 2025 Feb 17;16:e141288. doi: 10.3897/imafungus.16.141288 (PMC11882022; doi:10.3897/imafungus.16.141288)
Supplement: Supplementary material 2 — Supplementary images [file imafungus-16-e141288-s002.docx]

**

**

**Figure S1**. Putative secondary structures of tRNA genes identified in the mitochondrial genomes of *Daedaleopsis sinensis*. The tRNAs in green or red fonts represent tRNAs shared by the three *Daedaleopsis* species, while the tRNA in blue font represents tRNA found only in *D. nitida*. Residues conserved across the three mitochondrial genomes are shown in green, while variable sites are shown in red. All genes are shown in their orders of occurrence in the mitochondrial genome of *D. sinensis* starting from *trnS*.

**
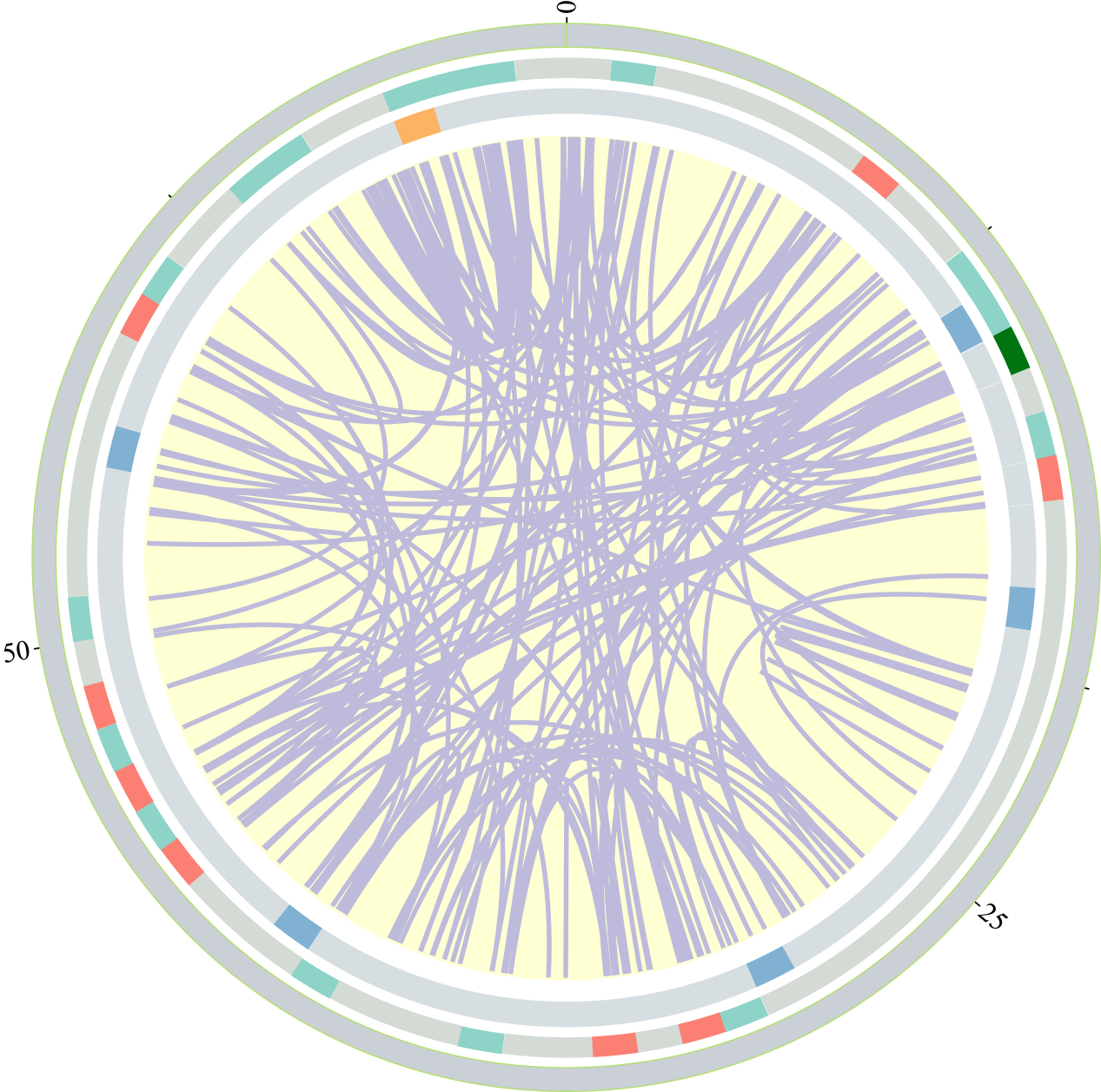
**

**Figure S2**. Distribution of repeats in the mitochondrial genome of *Daedaleopsis sinensis*. The outermost circle represents the SSRs, followed by the tandem repeats, while the lines in the innermost circle represent interspersed repeats.

**
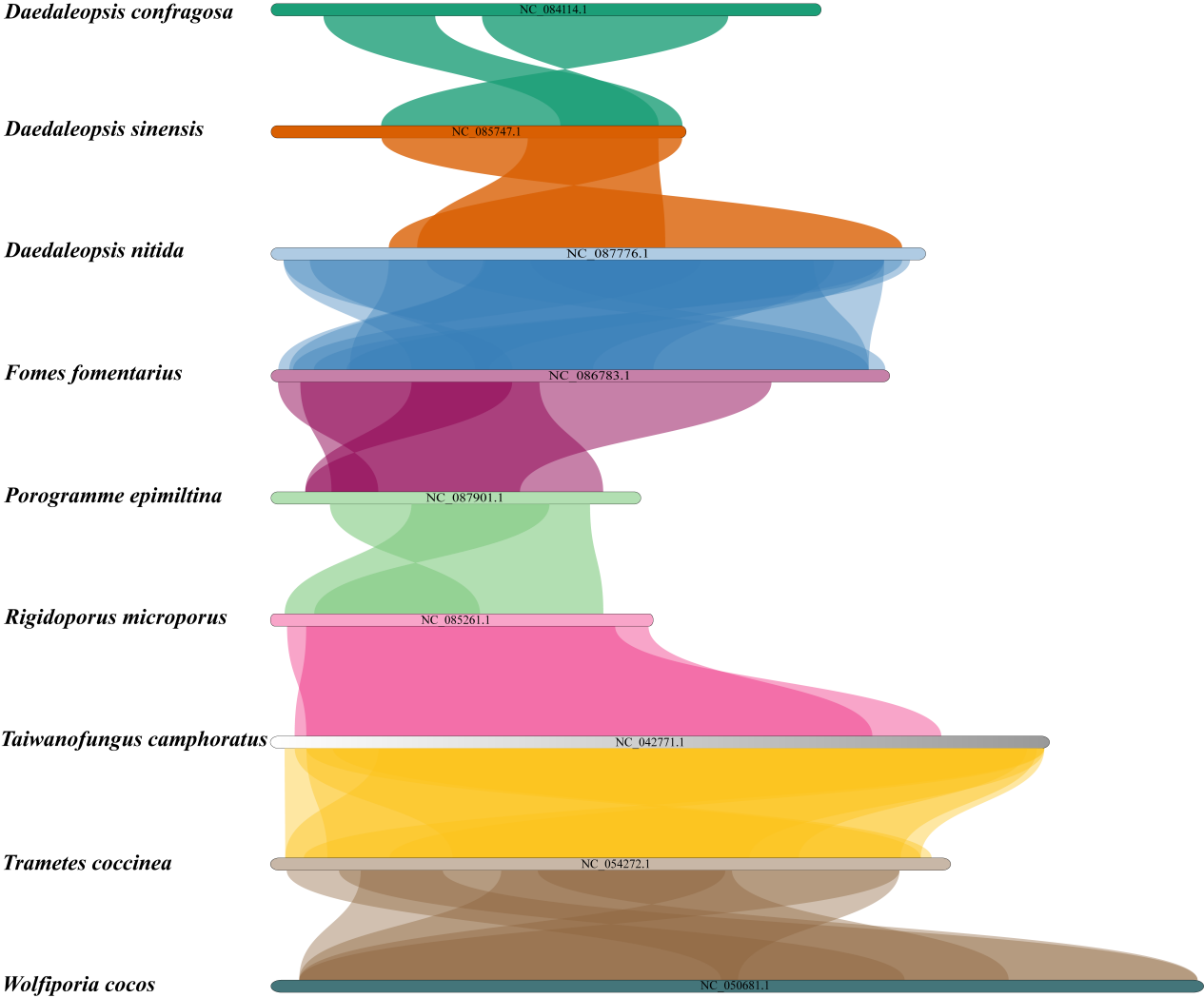
**

**Figure S3**. Collinearity analysis amongst the nine mitochondrial genomes. Bars represent the mitochondrial genomes, while ribbons indicate the homologous sequences between adjacent species.

**
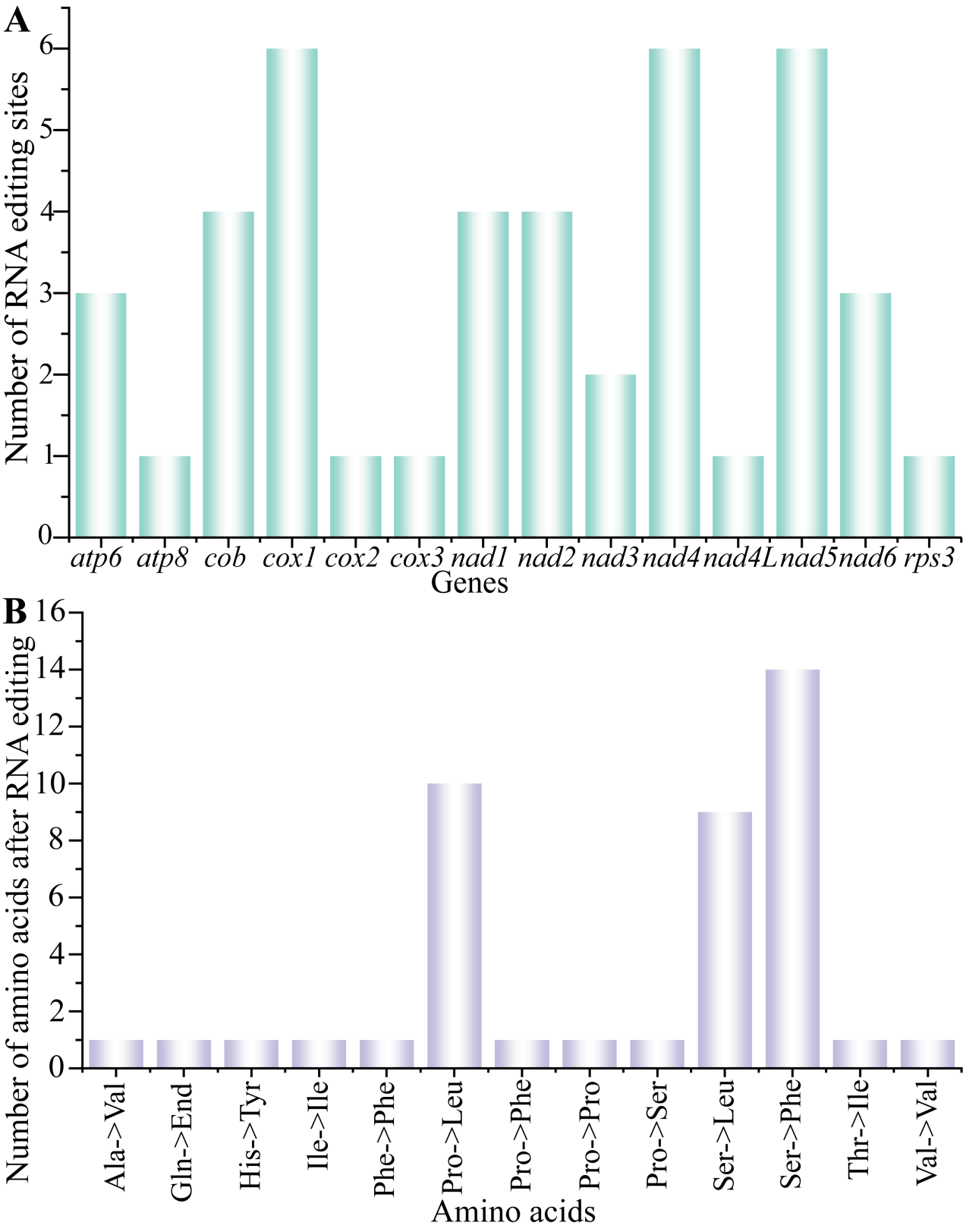
**

**Figure S4**. Characteristics of the RNA editing sites identified in the mitochondrial PCGs of *Daedaleopsis sinensis*. **A** Diagram of the distribution of RNA editing sites in the mitochondrial PCGs of *D. sinensis*. Bars depict the number of RNA editing sites for each gene; **B** Statistics on the potential effects of RNA editing events. The x-axis represents the changes in amino acids, while the y-axis represents the number of times each change occurred.
